# Comparison of Unicompartmental Knee Arthroplasty Versus High Tibial Osteotomy for Medial Knee Osteoarthritis: An Updated Meta‐Analysis of 56,000 Patients

**DOI:** 10.1111/os.70049

**Published:** 2025-07-22

**Authors:** Muhammad Hassan Waseem, Zain ul Abideen, Muhammad Haris Khan, Muhammad Fawad Tahir, Muhammad Mukhlis, Aisha Kakakhail, Eiman Zeeshan, Mahnoor Usman, Misha Khalid, Ameer Haider Cheema, Sania Aimen, Javed Iqbal, Haseeb Javed Khan

**Affiliations:** ^1^ Allama Iqbal Medical College Lahore Pakistan; ^2^ King Edward Medical University Lahore Pakistan; ^3^ Saidu Medical College Swat Pakistan; ^4^ H.B.S Medical and Dental College Islamabad Pakistan; ^5^ Ayub Medical College Abbottabad Pakistan; ^6^ Khyber Girls Medical College Peshawar Pakistan; ^7^ Dow Dental College Karachi Pakistan; ^8^ Karachi Medical and Dental College Karachi Pakistan; ^9^ UT Southwestern Medical Center Dallas Texas USA; ^10^ Quetta Institute of Medical Sciences Balochistan Pakistan; ^11^ Nursing Department Hamad Medical Corporation Doha Qatar

**Keywords:** high tibial osteotomy, meta‐analysis, osteoarthritis, unicompartmental knee arthroplasty

## Abstract

Osteoarthritis (OA) is a prevalent degenerative joint disease primarily affecting hip and knee joints, with an estimated 300 million cases globally. This study is crucial as it provides an updated, comprehensive comparison of unicompartmental knee arthroplasty (UKA) and high tibial osteotomy (HTO) for treating medial knee osteoarthritis, offering valuable insights into their relative effectiveness. The findings aim to inform clinical decision‐making and improve patient outcomes by identifying the superior treatment option. A comprehensive search was conducted across PubMed, Cochrane Library, and Google Scholar until August 1, 2024. Statistical analysis used Review Manager 5.4 with a random‐effects model, risk ratio (RR), and mean differences (MD) with 95% confidence intervals (CI) for the dichotomous and continuous outcomes, respectively. The Newcastle‐Ottawa Scale was used for quality assessment, and funnel plots were used to analyze publication bias. GRADE assessment was done to gauge the certainty of the evidence. Thirty‐nine studies, involving a total of 56,686 patients, were evaluated for comparison. UKA significantly reduced the complications (RR = 0.37; 95% CI: [0.25, 0.54]; *p* < 0.0001; I^2^ = 30%), revision rates to total knee arthroplasty (TKA) (RR = 0.64; 95% CI: [0.41, 0.99]; *p* = 0.05; I^2^ = 72%) and postoperative pain (MD = −0.33; 95% CI: [−0.64, −0.03]; *p* = 0.03; I^2^ = 89%) compared to HTO, while range of motion (ROM) (RR = −3.55; 95% CI: [−7.16, 0.52]; *p* = 0.09; I^2^ = 98%) and walking speed (MD = 0.02; 95% CI: [−0.04, 0.07]; *p* = 0.56; I^2^ = 0%) and surgical site infections(RR = 1.40; 95% CI: [0.30, 6.53]; *p* = 0.67; I^2^ = 86%) were comparable. All the functional knee scores are comparable except the Hospital for Special Surgery (HSS) score, which is increased in UKA (MD = 2.63; 95% CI: [0.52, 4.74]; *p* = 0.01; I^2^ = 76%). UKA is superior to HTO, offering lower revision rates, reduced postoperative pain, fewer complications, and better functional scores.

## Graphical abstract

Unicompartmental knee arthroplasty (UKA) is superior to high tibial osteotomy (HTO) for patients with medial knee osteoarthritis, offering fewer complications, lower revision rates, reduced postoperative pain, and a comparable range of motion.

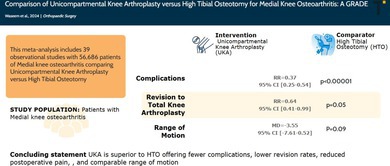

## 1 Introduction

Osteoarthritis (OA) is a persistent degenerative joint disease characterized by underlying bone and cartilage deterioration [1]. Although OA can affect different joints, most social OA burdens are associated with hip and knee OA, and it has been estimated that there are 300 million cases of hip and knee OA globally [2]. While OA can affect any one or all three knee compartments, research has shown that 10%–29.5% of cases with arthritic change occur mostly in the medial compartment [3].

Both unicompartmental knee arthroplasty (UKA) and High tibial osteotomy (HTO) are the recommended treatments for unicompartmental knee OA [4]. HTO consists of cutting and reshaping bone to shift away the strain from the injured area of the knee to the unaffected compartment using various techniques, including open‐wedge HTO, close‐wedge HTO, and dome osteotomies [5, 6].UKA, also known as partial knee replacement, entails the resurfacing procedure in which a prosthetic implant is used to replace just the damaged compartment while the healthy part is kept preserved [5]. UKA typically produces superior functional outcomes and lower pain levels compared to HTO. Patients undergoing UKA recover frequently and require less time for therapy [7]. Since HTO tends to offer a little greater range of motion (ROM) than UKA [8], HTO is typically recommended for younger patients and allows for greater physical activity, while UKA is more suitable for older patients [7]. Moreover, UKA is associated with fewer complications than HTO. Yet, UKA can potentially see a positive reaction to its work with further development in surgical methods and the quality of implants.

A previous meta‐analysis comparing UKA and HTO for osteoarthritis (OA) yielded extensive results [3].Since the publication of the original meta‐analysis, several new studies have emerged on this topic [5, 9, 10, 11], potentially altering the overall effect size and influencing our understanding of the phenomenon. This updated meta‐analysis synthesizes the most recent evidence to provide a more comprehensive and current understanding. In our study, we aimed to assess whether there has been any shift in the significance of the key outcomes. This updated analysis highlights the comparison between UKA and HTO for OA, providing valuable evidence to support guideline‐directed medical treatment.

## 2 Methodology

We performed a systematic review and meta‐analysis per the guidelines set forth by the Preferred Reporting Items for Systematic Reviews and Meta‐Analyses (PRISMA) [9] and the Cochrane Handbook for Systematic Reviews of Interventions [10]. The protocol for this review has been pre‐registered on PROSPERO with the registration number CRD42024576504.

### 2.1 Search Strategy and Databases

A thorough electronic search was performed across three databases—PubMed, ScienceDirect, and Cochrane Library‐ from 1986 until August 1, 2024. The focus was on studies involving human subjects that compared the outcomes of UKA with HTO in patients diagnosed with Medial Knee Osteoarthritis. The following MeSH terms were utilized: “Arthroplasty, Replacement, Knee,” “Osteoarthritis,” “Osteotomy,” and “Tibia.” The detailed search strategy is outlined in Table S1.

### 2.2 Study Selection and Eligibility Criteria

**FIGURE 1:**
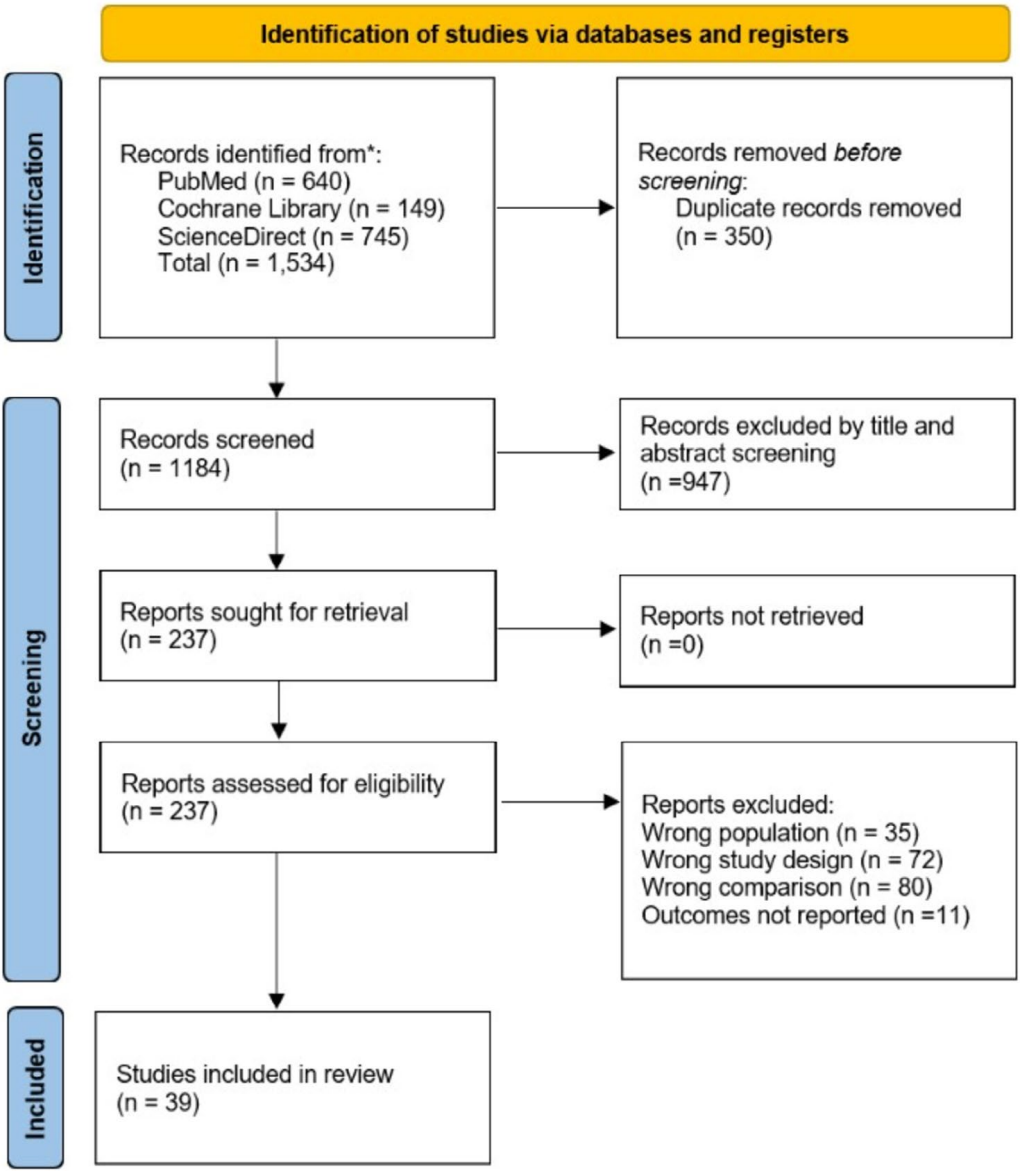
PRISMA flowchart of the study selection process.

The studies identified from the search were first deduplicated using EndNote. Following this, a two‐step screening process was implemented. First, the titles and abstracts were reviewed, followed by a detailed full‐text evaluation of the relevant studies. The screening was done independently by two reviewers (H.W. and Z.U.A). Eligible studies included both retrospective and prospective analyses that directly compared postoperative outcomes between UKA and HTO. Studies reporting at least one relevant outcome for comparison between Knee Arthroplasty and High Tibial Osteotomy were also considered. Exclusion criteria were: (i) insufficient data or abstract‐only publications, (ii) single‐arm studies, and (iii) publications such as letters, case reports, and systematic or narrative reviews. In this study, the effects of computer‐aided or robotic navigation on the study indicators, such as complication rates, revision rates, range of motion, postoperative pain, and functional scores, were not considered due to the limited availability of relevant data. Future studies with more comprehensive data are needed to assess the potential impact of these advanced technologies on surgical outcomes and to provide a more thorough understanding of their role in Unicompartmental Knee Arthroplasty (UKA) and High Tibial Osteotomy (HTO). The detailed study selection process is depicted in Figure 1.

### 2.3 Data Extraction and Outcomes

Two researchers (M.U. and M.H.K.) independently extracted data from the selected studies to ensure accuracy and minimize bias. After extraction, the data were carefully organized and recorded using Microsoft Excel for quantitative information and Microsoft Word for textual and descriptive details. The following outcomes were included in the analysis: (i) Complications, (ii) Revision to TKA, (iii) ROM, (iv) Postoperative pain (measured by the Visual Analog Scale [VAS]), (v) Lysholm score, (vi) Hospital for Special Surgery (HSS) score, (vii) Oxford Knee Score, (viii) Tegner score, (ix) Walking speed, (x) Western Ontario and McMaster Universities Osteoarthritis Index (WOMAC) score, and (xi) Knee Society Score (KSS). Baseline characteristics extracted from studies included: (i) Study period, (ii) Institution, (iii) Study design, (iv) Country, (v) Sample size (patients and knees), (vi) Mean age (in years, with standard deviation), (vii) Gender distribution (F/M), (viii) Body Mass Index (BMI in kg/m^2^, with standard deviation), (ix) Osteoarthritis severity (Kellgren‐Lawrence [K/L] and Ahlbäck [AH] grade), (x) Follow‐up duration (in months), (xi) Type of HTO (open or closed wedge), and (xii) Type of UKA (fixed or mobile bearing).

### 2.4 Risk of Bias

Two reviewers independently (M.K. and M.M.) evaluated the quality of the included studies, with any disagreement resolved by the Third author (E.Z.). The Newcastle–Ottawa scale, which assesses studies based on selection, comparability, and outcome, was used to assess the risk of bias for non‐randomized comparative studies [12]. The publication bias was assessed through funnel plots and Egger's regression. The GRADE assessment was performed to gauge the certainty of evidence by GRADEpro GDT [13].

### 2.5 Statistical Analysis

Statistical analysis was conducted using Review Manager version 5.4.1 [14]. A random‐effects model was applied to compute summary estimates for meta‐analysis. The risk ratio (RR) was calculated for dichotomous outcomes, while mean differences (MD) were used for continuous outcomes. Results were presented with 95% confidence intervals (CI). Heterogeneity was considered significant when I^2^ exceeded 50% and was evaluated using Higgins I^2^ statistics [15]. A *p*‐value of less than 0.05 was deemed statistically significant for all analyses. Subgroup and leave‐one‐out sensitivity analyses were conducted to identify possible sources of heterogeneity.

## 3 Results

### 3.1 Search Results

A detailed search was conducted across electronic databases, including PubMed (*n* = 640), Cochrane Library (*n* = 149), and ScienceDirect (*n* = 745), yielding a total of 1534 articles. 1184 articles were screened through title and abstract after removing duplicates (*n* = 350). Studies retrieved after the title and abstract screening (*n* = 237) were passed through the full‐length article screening, yielding 39 articles [5, 8, 9, 10, 11, 16, 17, 18, 19, 20, 21, 22, 23, 24, 25, 26, 27, 28, 29, 30, 31, 32, 33, 34, 35, 36, 37, 38, 39, 40, 41, 42, 43, 44, 45, 46, 47, 48, 49] that were included in the final quantitative analysis.

### 3.2 Characteristics of Included Studies

This review includes 39 studies [5, 8, 9, 10, 11, 16, 17, 18, 19, 20, 21, 22, 23, 24, 25, 26, 27, 28, 29, 30, 31, 32, 33, 34, 35, 36, 37, 38, 39, 40, 41, 42, 43, 44, 45, 46, 47, 48, 49] encompassing 56,686 patients published between 1986 and 2024. The sample size ranged from 10 to 23,563, with a mean age of 47 to 80 years and a mean BMI of 23 to 33 kg/m2. Eight studies [16, 17, 18, 19, 20, 21, 22, 34] reported the closed wedge HTO, whereas 29 studies [5, 8, 9, 10, 11, 23, 24, 25, 26, 27, 29, 30, 31, 32, 33, 35, 36, 37, 38, 39, 40, 41, 42, 43, 44, 45, 46, 48, 49] reported the open wedge HTO, with two studies [28, 47] reporting both the closed and open wedge HTO. Nineteen studies [5, 8, 9, 10, 11, 16, 18, 19, 20, 21, 22, 23, 24, 27, 28, 34, 39, 44, 45] reported fixed‐bearing UKA, 11 studies [17, 25, 26, 29, 36, 37, 40, 43, 46, 48, 49] reported mobile‐bearing UKA, and 1 study [47] reported both fixed and mobile‐bearing UKA. The details are provided in Table 1.

**TABLE 1:** Baseline characteristics of the included studies.

Authors name	Study design	Country	Sample size patients (knees)	Mean age in years (SD)	Gender F/M	BMI kg/m2 (SD)		OA severity (K/L grade)	Follow‐up duration (months)	Type of HTO (open wedge or close wedge)	Type of UKA (Fixed or mobile bearing)
						UKA	HTO				
Broughton 1986	RC	England	91	71	38/11 HTO 31/11 UKA	—	—	—	26.3 ± 18 HTO 69.6 ± 14.4UKA	CW	FB
Jefferson 1989	PC	UK	42	64	—	—	—	—	—	CW	MB
Ivarsson 1991	RC	Sweden	20	64 ± 5	6/4 HTO 6/4 UKA	—	—	—	6 HTO/12 UKA	CW	FB
Weidenhielm 1992	PC	Sweden	53	63	14/11 HTO 14/14 UKA	—	—	—	12	CW	FB
Weale 1994	RC	England	91	80	—	—	—	—	144 to 204	CW	FB
Stukenborg 2001	PC	Germany	62	67	13/19 HTO 22/6 UKA	—	—	—	84 to 120	CW	FB
Borjesson 2005	PC	Sweden	40	63 ± 4	8/10 HTO 11/11 UKA	—	—	—	60	CW	FB
Takeuchi 2010	RC	Japan	57	77 ± 4	18/6 HTO 14/4 UKA	—	—	—	61 ± 10 HTO 84 ± 4 UKA	OW	FB
Yim 2013	RC	South Korea	108	57.61 ± 4.783 HTO 59.54 ± 4.012 UKA	51/7 HTO 48/2 UKA	—	—	K‐L grade ≥ 2 OA	43.2 HTO 44.4 UKA	OW	FB
Tuncay 2015	RC	Turkey	202	58.7	70/18 HTO 79/15 UKA	—	—	—	36 HTO 42 UKA	OW	MB
Petersen 2016	RC	Germany	48	60.7	9/14 HTO 16/9 UKA	25	23	—	60 HTO 60 UKA	OW	MB
Jeon 2017	RC	Korea	47	56.85 ± 5.55	22/4 HTO 17/4 UKA	26.1	26.6	K–L grade ≥ 3 OA	24 HTO 24 UKA	OW	FB
Krych 2017	RC	USA	240	49.2	16/41 HTO 101/82 UKA	32.4	31.8	K‐L grade 2 HTO/3UKA	86.4 HTO 69.6 UKA	OW + CW	FB
Maxwell 2017	RC	New Zealand	170	> 55	0/75 HTO, 0/95 UKA	—	—	—	97.2 HTO 73.2 UKA	OW	MB
Zhao 2017	PC	China	72	52.47 ± 8.41	33/3 HTO 31/5 UKA	—	—	K–L grade 2/3 10/26 HTO 11/25 UKA	24 HTO 12 UKA	OW	NR
Cho 2018	RC	South Korea	40	67.9 ± 9.0	12/8 HTO 19/1 UKA	26.1 ± 2.6	26.5 ± 2.5	AH grade 2/3 18/2 HTO 13/7 UKA	48.4 ± 14.3 HTO 39.7 ± 14.0 UKA	OW	NR
Ryu 2018	RC	Korea	45	60.5 ± 3.4	22/1 HTO 19/3 UKA	25.4 ± 3.6	27.7 ± 2.9	—	40.0 ± 19.4 HTO 33.1 ± 8.7 UKA	OW	NR
Koh 2019	RC	Korea	241	60.8 ± 4.7	104/19 HTO 98/20 UKA	25.8 ± 2.9	25.9 ± 3.2	AH grade < 2/≥ 2 97/26 HTO 37/81 UKA	24 HTO 24 UKA	OW	NR
Song 2019	RC	South Korea	110	60.8 ± 3.9	51/9 HTO 43/7 UKA	25.3 ± 3.4	25.1 ± 3.6	K–L grade ¾	128.4 ± 68.4 HTO 144 ± 85.2 UKA	CW	FB
Jacquet 2020	RC	France	100	50.8 ± 4.4	22/28 HTO 21/29 UKA	27.1 ± 3.1	26.6 ± 2.6	—	44.4 ± 19.2 HTO 46.6 ± 21.6 UKA	OW	NR
Chen 2020	RC	—	38	56.1 ± 6.5	13/5 HTO 14/6 UKA	—	—	K–L grade 2/3	12–33.6 HTO 12–38.4 UKA	OW	MB
Hou 2020	RC	China	60	—	—	—	—	—	—	OW	MB
Zhang 2020	RC	China	192	51.8 ± 6.9	86/23 HTO 66/17 UKA	27.7 ± 4.1	26.4 ± 3.6	—	40.2 ± 13.5 HTO 39.3 ± 11.2 UKA	OW	NR
Jin 2021	RC	South Korea	134	63.1 ± 4.9	67/0 HTO 65/2 UKA	25.5 ± 2.8	25.5 ± 3.1	K–L grade ¾	> 12	OW	FB
Lin 2021	RC	—	114	61.4 ± 4.7	40/13 HTO 46/15 UKA	26.0 ± 3.1	26.3 ± 3.2	K–L grade 2/3	> 12	OW	MB
Rodkey 2021	RC	USA	383	48	12/101 HTO 80/190UKA	31	29.8	—	63.6 HTO 75.6 UKA	OW	NR
Watanabe 2021	RC	Japan	96	73.8 ± 5.2	—	24.1 ± 2.8	26.1 ± 3.8	K‐L grade 2/3	24.0 HTO 22.5 UKA	OW	NR
Liu 2021	RC	NR	97	61.2 ± 2.8	32/16 HTO 31/18HTO	27.3 ± 2.1	28.1 ± 1.8	K‐L grade 2/3	39.6 HTO 42 UKA	OW	MB
Wyatt 2024	PC	UK	42	54.3 ± 10.8 HTO 66.9 ± 8.6 UKA	1/18 HTO 11/12UKA	30.0 ± 4.6	31.5 ± 4.8	—	12	OW	FB
Screpis 2023	RC	Italy	620	> 50	18F HTO 19F UKA	27.86 ± 2.05	28 ± 1.67	KL grade 2/3	6	OW	FB
Teo 2024	RC	Singapore and Indonesia	203	56.42 ± 6.07 HTO 58.57 ± 5.15 UKA	30/17HTO, 32/15UKA	29.09 ± 4.98	29.82 ± 5.06	KL grade ¾	24	OW	FB
Hoorntje 2023	RC	Netherlands	214	55.0 ± 3.0 HTO 55.7 ± 2.8 UKA	66M HTO/61M UKA	28.7 ± 4.0	27.8 ± 3.4	KL grade ¾	6, 12 and 24	OW	FB
Zehir 2022	RC	Turkey	105	56.35 ± 3.60 HTO 56.90 ± 3.80 UKA	—	28.97 ± 4.01	26.79 ± 4.21	KL grade 2/> 2	10 ± 8.14 (5 years)	OW	FB
Xu 2024	RC	China	68	58.66 ± 2.74 HTO 59.50 ± 3.70 UKA	14/22 HTO 18/14 UKA	23.76 ± 3.08	24.77 ± 3.05	KL grade 3	18	OW	MB
Lee 2021	RC	Korea	47,126	56.53 ± 7.60 HTO 63.81 ± 8.39 UKA	45,536/15,609 HTO 24,184/5376 UKA	—	—	—	138	OW + CW	FB + MB
Neubauer 2023	RC	Austria	86	57.41 ± 0.7267 UKA 52.017 ± 1.1972 HTO	6/19HTO 40/21UKA	30.23 ± 0.705	28.84 ± 1.008	—	77.13 (±8.170)	OW	FB
Ozgozen 2024	RC	Turkey	208	55.9 ± 3.9 HTO 57.2 ± 4.9 UKA	45/7HTO 78/9 UKA	27.9 ± 2	28.3 ± 1.6	KL grade 2/3	135.6	OW	MB
Karasavvidis 2024	RC	USA	4911	46.6 ± 8.4 HTO 53.46 ± 5.5 UKA	45F HTO 2535 F UKA	33.26 ± 6.6	31.7 ± 6 5.9	—	1	OW	FB
Okimura 2023	RC	Japan	110	69.1 ± 2.6 HTO 70.1 ± 2.9 UKA	58/15 HTO 25/12 UKA	27.6 ± 4.6	26.6 ± 3.4	K/L grades 2, 3, 4	60	OW	MB

Abbreviations: AH grade, Ahlbäck grade; CW, Close Wedge; FB, Fixed Bearing; HTO, High Tibial Osteotomy; K/L grade, Kellgren‐Lawrence grade; MB, Mobile Bearing; OA, Osteoarthritis; OW, Open Wedge; PC, Prospective cohort; RC, Retrospective cohort; SD, Standard deviation; UKA, Unicompartmental Knee Arthroplasty.

### 3.3 Risk of Bias and GRADE Assessment

The NOS [50] was used for the observational cohort's quality assessment. The NOS details are presented in Table S2. We used the GRADE assessment, as detailed in Table 2, to evaluate the certainty of the evidence.

**TABLE 2:** GRADE assessment of certainty of evidence.

Patient or population: Medial knee osteoarthritis					
Intervention: UKA					
Comparison: HTO					
Outcomes	**Anticipated absolute effects** (95% CI)		Relative effect (95% CI)	№ of participants (studies)	Certainty of the evidence (GRADE)
	**Risk with HTO**	**Risk with UKA**			
Complications	95 per 1000	**35 per 1000** (24 to 51)	**RR 0.37** (0.25 to 0.54)	7719 (25 studies)	⨁⨁◯◯ Low
Revision to TKA	61 per 1000	**39 per 1000** (25 to 60)	**RR 0.64** (0.41 to 0.99)	49,687 (20 studies)	⨁◯◯◯ Very low^a^
Range of motion (ROM)	The mean range of motion (ROM) ranged from **99**° to **149**°	MD **3.55° lower** (7.61 lower to 0.52 higher)	—	1141 (14 studies)	⨁⨁◯◯◯ Very low^**a**^, ^**b**^
Postoperative pain visual analog scale (VAS) score	The mean postoperative pain visual analog scale (VAS) score ranged from **0.5 to 3.5**	MD **0.33 lower** (0.64 lower to 0.03 lower)	—	900 (9 studies)	⨁◯◯◯ Very low^a^
Lysholm score	The mean Lysholm score ranged from **78 to 96**	MD **1.8 higher** (0.85 lower to 4.44 higher)	—	807 (9 studies)	⨁◯◯◯ Very low^**a**^, ^**b**^
HSS score	The mean HSS score ranged from **82 to 94**	MD **2.63 higher** (0.52 higher to 4.74 higher)	—	695 (8 studies)	⨁◯◯◯ Very low^a^
KSS score	The mean KSS score ranged from **15 to 89**	MD **0.68 lower** (5.29 lower to 3.93 higher)	—	520 (6 studies)	⨁◯◯◯ Very low^**a**^, ^**b**^
WOMAC score	The mean WOMAC score ranged from **13 to 33**	MD **4.33 lower** (10.56 lower to 1.91 higher)	—	530 (4 studies)	⨁◯◯◯ Very low^**a**^, ^**b**^
Tegner score	The mean Tegner score ranged from **2.5 to 5**	MD **0.07 higher** (0.37 lower to 0.5 higher)	—	544 (6 studies)	⨁◯◯◯ Very low^**a**^, ^**b**^
Oxford knee score (OKS)	The mean Oxford knee score (OKS) ranged from **18 to 43**	MD **0.28 higher** (1.25 lower to 1.81 higher)	—	489 (4 studies)	⨁◯◯◯ Very low^b^
Surgical site wound infection	9 per 1000	**13 per 1000** (3 to 59)	**RR 1.40** (0.30 to 6.53)	52,500 (6 studies)	⨁◯◯◯ Very low^**a**^, ^**c**^
Walking speed	The mean walking speed ranged from **0.99 to 1.35**	MD **0.02 higher** (0.04 lower to 0.07 higher)	—	148 (4 studies)	⨁◯◯◯ Very low^b^

*Note*: The bold text is just standard text formatting style for grade table it doesn't specifiy anything.

Abbreviations: CI, confidence interval; MD, mean difference; RR, risk ratio.

^**a**^ Heterogeneity is > 60%.

^**b**^ 95% Confidence Interval is crossing the 0.

^**c**^ 95% Confidence Interval is crossing the 1.

### 3.4 Outcomes

#### 3.4.1 Complications

**FIGURE 2:**
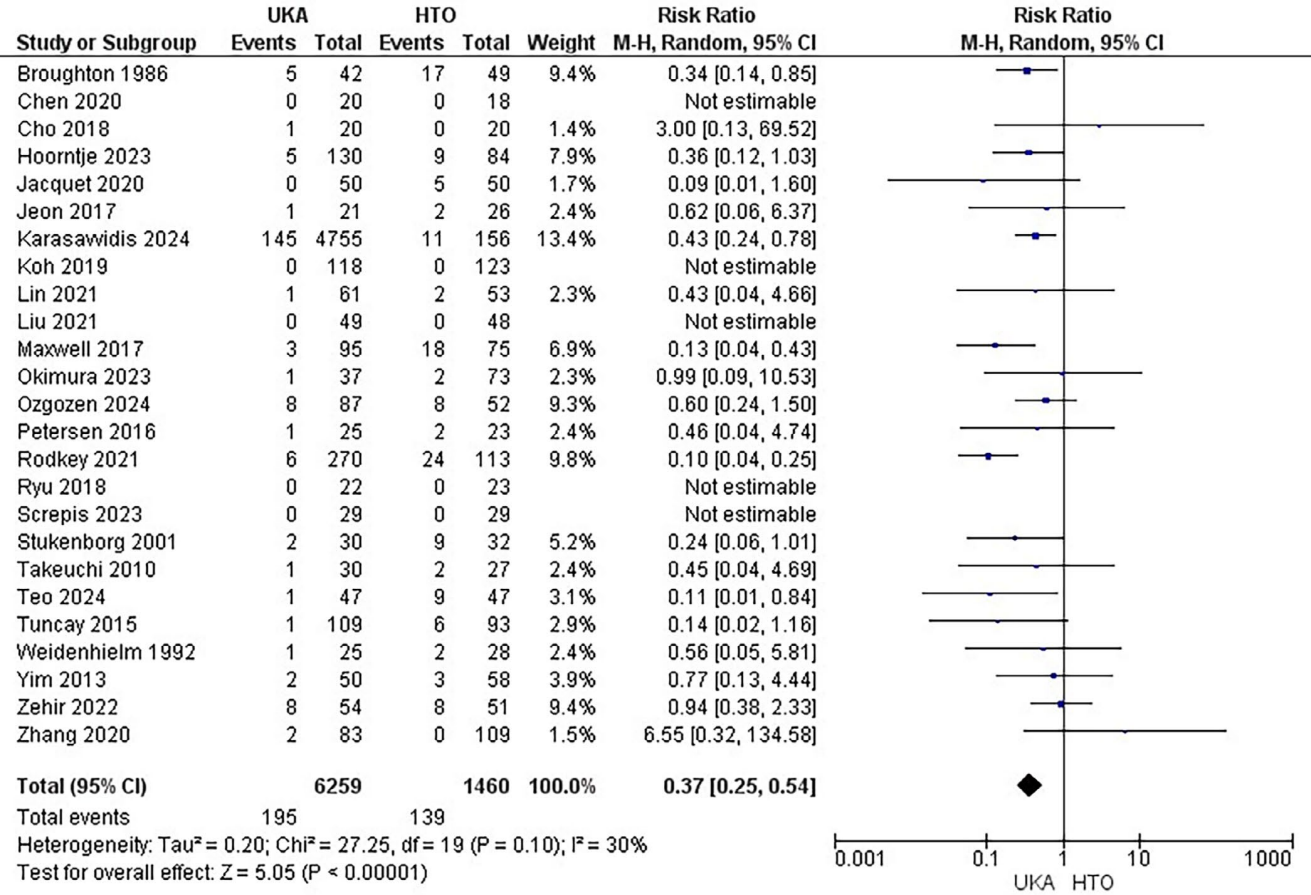
Complications Forest plot.

Complications were reported by 25 studies [5, 9, 11, 16, 19, 20, 23, 24, 25, 26, 27, 29, 31, 32, 33, 35, 36, 38, 40, 41, 43, 44, 45, 48, 49] comprising a total of 7719 patients (6259 UKA versus 1460 HTO). UKA decreased the complications compared to the HTO (RR = 0.37; 95% CI: [0.25, 0.54]; *p* < 0.00001; I^2^ = 30%) Figure 2. On subgroup analysis, UKA was associated with a lower risk of complications in the retrospective studies group (RR = 0.38, 95% CI: [0.25, 0.57]; *p* < 0.00001; I2 = 36%) while the results were statistically non‐significant in the prospective studies group (RR = 0.30, 95% CI: [0.09, 1.03]; *p* = 0.06; I2 = 0%) (Figure S16).

#### 3.4.2 Revision to Total Knee Arthroplasty

**FIGURE 3:**
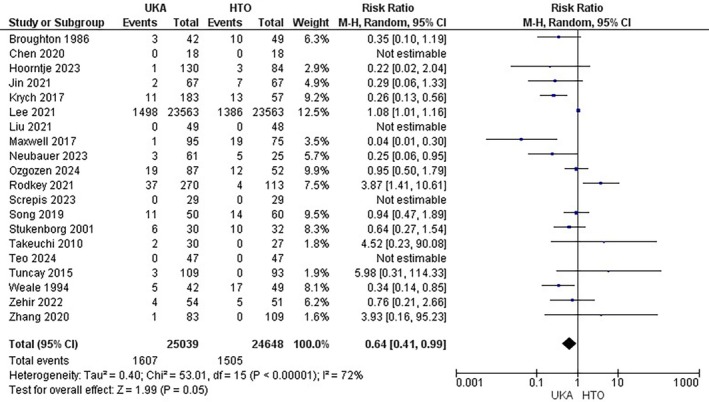
Revision to Total Knee Arthroplasty Forest plot.

Twenty studies [8, 9, 11, 16, 20, 21, 23, 25, 28, 29, 34, 36, 38, 39, 41, 43, 44, 45, 47, 48] comprising a total of 49,687 patients (25,039 UKA versus 24,648 HTO) who reported the revision to TKA. UKA decreased the revision compared to HTO with a pooled RR of 0.64 (95% CI: [0.41, 0.99]; *p* < 0.05). The overall heterogeneity was high, I^2^ = 72% Figure 3.

#### 3.4.3 Range of Motion

**FIGURE 4:**
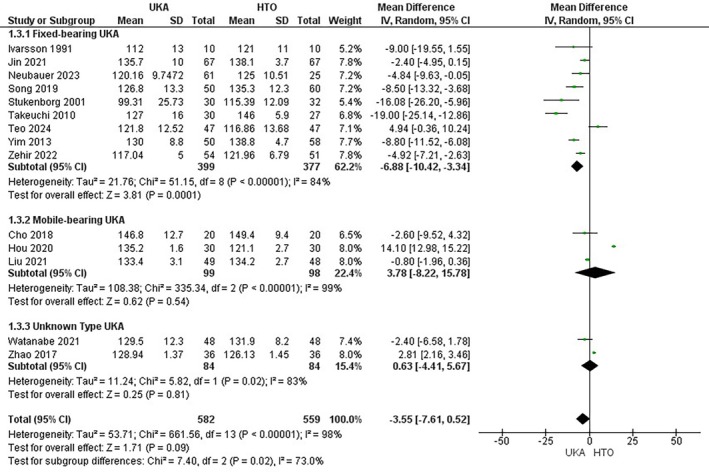
Range of Motion Forest plot.

Fourteen studies [8, 11, 18, 20, 23, 24, 30, 31, 34, 37, 39, 42, 43, 45] encompassing 1141 patients (582 UKA versus 559 HTO) were included in the quantitative pooling of the ROM outcome. The ROM was comparable between both groups (MD = −3.55; 95% CI: [−7.16, 0.52]; *p* < 0.09; I^2^ = 98%). On subgroup analysis according to the type of UKA, the results remained insignificant for the mobile‐bearing (MD =3.78; 95% CI:[−8.22, 15.78]; *p* < 0.54; I^2^ = 99%) and unknown (MD =0.63; 95% CI:[−4.41, 5.67]; *p* < 0.81; I^2^ = 83%) UKA groups, except for the fixed‐bearing (MD = ‒6.88; 95% CI:[−10.42,‐3.34]; *p* < 0.0001; I^2^ = 84%) UKA, which significantly decreased the ROM (Figure 4).

#### 3.4.4 Postoperative Pain

**FIGURE 5:**
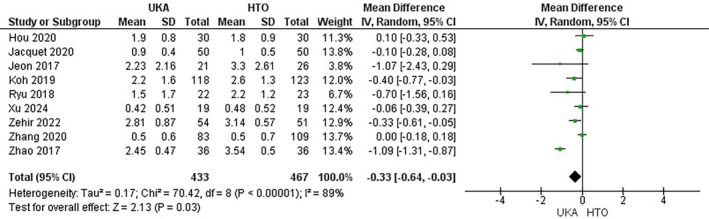
Postoperative pain Forest plot.

Postoperative pain was reported by 9 studies [27, 30, 32, 33, 35, 37, 38, 45, 46] encompassing 900 patients (433 UKA versus 467 HTO). VAS score, an indicator of postoperative pain, was found to be lower in the UKA group compared to the HTO group (MD = −0.33; 95% CI: [−0.64, −0.03]; *p* = 0.03; I^2^ = 89%) (Figure 5).

#### 3.4.5 Surgical Site Wound Infection

Six studies [5, 24, 38, 44, 45, 47] reported surgical site wound infection, comprising a total of 52,500 patients (28,534 UKA versus 23,966 HTO). Both study groups were comparable regarding this outcome (RR = 1.4; 95% CI: [0.30, 6.53]; *p* = 0.67; I^2^ = 86%) Figure S1.

#### 3.4.6 Walking Speed

Walking speed is reported by 4 studies [17, 18, 19, 22] comprising a total of 148 patients (72 UKA versus 76 HTO). The walking speed was comparable between the two groups (MD = 0.02; 95% CI: [−0.04, 0.07]; *p* = 0.56; I^2^ = 0%) Figure S2. On subgroup analysis, the two surgical approaches were also comparable in the retrospective studies (MD = −0.15, 95% CI: [−0.45, 0.15]; *p* = 0.33) and prospective studies group (MD = 0.02, 95% CI: [−0.03, 0.08]; *p* = 0.43, I2 = 0%) respectively (Figure S17).

#### 3.4.7 Functional Knee Scores

Different scores were used to compare the postoperative functional outcomes between the UKA and HTO groups. Eight studies [30, 31, 32, 37, 38, 39, 40, 46] comprising 695 patients reported a higher HSS score in the UKA group compared to HTO (MD = 2.63; 95% CI: [0.52, 4.74]; *p* = 0.01; I^2^ = 76%) (Figure S3).

The KSS score functional knee score was comparable between UKA and HTO (MD = −0.68; 95% CI: [−5.29, 3.93]; *p* = 0.77; I^2^ = 88%) (Figure S4). On subgroup analysis, the two approaches were also comparable in the retrospective studies (MD = ‒0.43, 95% CI: [−5.55, 4.69]; *p* = 0.87; I^2^ = 90%) and prospective studies group (MD = ‒2.10, 95% CI: [−10.22, 6.02]; *p* = 0.61) respectively (Figure S18).

There was no significant difference in Oxford knee score (MD = 0.28; 95% CI: [−1.25, 1.81]; *p* = 0.72; I^2^ = 39%) between the two groups (Figure S6). On subgroup analysis, the two surgical approaches were comparable in retrospective studies (MD = 0.22, 95% CI: [−1.73, 2.18]; *p* = 0.82; I^2^ = 59%) and prospective studies (MD = 0.30, 95% CI: [−3.18, 3.78]; *p* = 0.87) respectively (Figure S19).

Other functional knee scores including Lysholm score (MD = 1.80; 95% CI:[−0.85, 4.44]; *p* = 0.18; I^2^ = 89%) (Figure S5), WOMAC score (MD = −4.33; 95% CI:[−10.56, 1.91]; *p* = 0.17; I^2^ = 86%) (Figure S7), and Tegner score (MD = 0.07; 95% CI:[−0.37, 0.50]; *p* = 0.77; I^2^ = 89%) (Figure S8)were also comparable between the two groups.

### 3.5 Sensitivity Analysis

For the outcomes with heterogeneity > 50%, we performed the leave‐one‐out sensitivity analysis. By removing the study by Zhao et al., the heterogeneity was reduced to 35% in the postoperative pain VAS score outcome [30] (Figure S9). Similarly, for the HSS score, the heterogeneity decreased to 23% upon removing the Zhao et al. study [30] Figure S10. On removing the study by Karasawidis et al. [5] the heterogeneity decreased to 0% for the surgical site infection (Figure S11).

### 3.6 Publication Bias

Upon visually examining the funnel plots and using the statistical Egger's regression test for outcomes pooling more than 10 studies, no significant publication bias was detected Figure S12–S15 and Table S3.

## 4 Discussion

### 4.1 Main Findings

This meta‐analysis of 39 observational studies evaluated the safety and effectiveness of UKA compared to HTO for medial knee osteoarthritis. The findings indicate that UKA significantly reduces the risk of complications, postoperative pain, and the need for revision to TKA while also demonstrating higher HSS scores. Although there is no substantial difference between UKA and HTO in terms of SSI, KSS score, Lysholm score, Oxford Knee Score, ROM, Tegner score, walking speed, or WOMAC score, UKA consistently shows a lower risk of conversion to TKA, reinforcing the credibility of our results. Factors such as aseptic loosening, fractures around the prosthesis, and infections contribute to revisions [51]. For patients with isolated medial compartment disease, UKA may offer a longer‐term solution due to its less invasive nature and preservation of healthy knee tissue.

The complication rates with HTO are proportionately higher than those with UKA. This evidence aligns with previous meta‐analyses that demonstrated the same results, contributing to the rationale that HTO is associated with increased complications compared to UKA [51, 52].The traditionally lower complication incidence in UKA is attributed to the procedure's minimally invasive nature. This often entails smaller incisions and diminished disturbance of surrounding tissues, resulting in expedited recovery times and decreased surgical trauma [5]. Moreover, UKA typically requires under 90 min, whereas HTO averages 112 min, leading to diminished complication rates [5]. Additionally, UKA is generally performed on elderly and less active patients, contingent upon proper selection, which may lead to reduced complication rates [53]. The reduced complication rate associated with UKA relative to HTO is attributable to the synergistic effects of a less invasive technique, shortened surgical duration, and stringent patient selection criteria.

Postoperative pain, measured on the VAS, which is regarded as the most determining factor of satisfaction, is considerably lower among patients undergoing UKA compared to those undergoing HTO. This result aligns with prior meta‐analyses that highlighted lower pain levels following UKA. A meta‐analysis revealed that HTO was linked to higher complication rates and poorer functional results, while UKA provided enhanced pain alleviation and greater overall patient satisfaction [54]. UKA can preserve more of the original anatomic structure and function of the knee and, therefore, improve pain management and shorten recovery time. The maintenance of function is primarily because of the less invasive aspects of UKA, which facilitate the retention of cruciate ligaments and adjacent soft tissues sometimes compromised by HTO. Research demonstrates that UKA enhances the maintenance of knee kinematics, allowing for instant full weight‐bearing and expedited mobilization post‐surgery, hence significantly enhancing recovery results [3].

Regarding the potential for SSI, the risk was comparable in both UKA and HTO. A systematic review and meta‐analysis performed by Huang et al. states that there is no difference in the rate of surgical site infection between UKA and HTO, thus concluding that both procedures carry similar risks in this regard [3]. No objective disagreement is presented on this conclusion by a study by Li, which thoroughly looked at the benefits of HTO and UKA and found that the surgical site infection rates were the same for the two alternatives [54]. Liu's meta‐analysis suggests that there is no added benefit offered by HTO over UKA and vice versa for patients with single‐compartment knee OA, as the complication rate with each of the procedures is comparable Liu [55]. The study conducted by Wyles et al. shows that the provision of a single dose of perioperative antibiotics in UKA is not associated with increased incidence of surgical site infection, which means that effective measures to reduce infection risk can be instituted [56].

### 4.2 Functional Outcomes

This meta‐analysis reveals noticeable differences in the functional outcomes of patients who have undergone UKA and HTO, particularly in terms of HSS scores. This data supports previous studies, which have shown statistically better HSS scores in UKA patients compared to HTO patients, suggesting that UKA may provide better pain control and recovery from disability [8, 52]. The comparative assessment of KSS, the Lysholm Score, the Oxford Knee Score, the Tegner Activity Score, and the WOMAC Score showed no significant effect of either procedure over the other. The KSS indicates that while UKA may perform better in certain technical parameters, it does not significantly exceed HTO in total knee function, as assessed by KSS [45, 52]. Likewise, the Lysholm and Oxford scores, which assess knee functionality and quality of life, showed no appreciable variations, suggesting that both treatments may generate comparable results in specific groups of patients [52, 57]. This trend was confirmed by the Tegner Activity Score and WOMAC scores, which showed no appreciable variations, therefore underlining the point that even though UKA could result in better HSS scores, it does not produce better outcomes for every single functional criterion [58].

A comparison of the dynamics of the ROM and walking speed after UKA and HTO seems to bring additional evidence to support this finding. The negligible variation in the range of motion suggests that the two procedures may yield comparable results in knee mobility post‐surgery. This outcome corresponds with recent studies indicating that while UKA generally produces better overall physiological results, the range of mobility achieved may not significantly differ from that achieved with HTO [59]. Takeuchi et al. noted that the ROM at 7–10 years after either surgery was slightly better after closed wedge HTO than after UKA, indicating the variability of the effect based on the type of surgery and the patients' factors [23]. Similarly, the analysis indicated that there seemed to be no statistically relevant difference in walking speed after UKA and HTO. This conclusion implies that either of the two approaches may result in comparable recovery in terms of speed of walking for patients who have undergone surgery on the knee joints, which is a major factor in patients' independence in daily routines post‐knee surgery [60, 61].

### 4.3 Limitations

We acknowledge several limitations in this meta‐analysis. First, the studies included were observational, which may introduce biases and limit the strength of our findings. Second, due to the limited data available, we were unable to assess the effects of computer‐aided or robotic navigation on the study indicators, including complication rates, revision rates, range of motion, and functional scores. Furthermore, significant heterogeneity was observed in several outcomes, which could impact the generalizability of the results. Lastly, the inclusion of studies over a long period (1986–2024) introduces variability due to advancements in surgical techniques and patient management. Further high‐quality randomized controlled trials (RCTs) are required to strengthen the evidence and draw definitive conclusions.

### 4.4 Conclusion

This meta‐analysis indicates that UKA is associated with a significantly lower risk of complications and the need for revisions to TKA when compared to HTO in patients with medial knee osteoarthritis. UKA is correlated with reduced postoperative pain levels and enhanced functional outcomes, as evidenced by HSS scores. Both procedures exhibit comparable rates of SSI and specific functional metrics; however, the minimally invasive nature of UKA is likely a contributing factor to its benefits in recovery duration and overall patient satisfaction. The results indicate that UKA could be the optimal choice for patients who meet specific selection criteria; however, additional studies are necessary to enhance patient selection processes and improve surgical methodologies.

## Author Contributions

Study concept and design: Muhammad Hassan Waseem and Zain ul Abideen. Acquisition of data: Zain ul Abideen and Muhammad Haris Khan. Analysis and interpretation of data: Muhammad Fawad Tahir and Muhammad Mukhlis. Drafting of the manuscript: Aisha Kakakhail, Eiman Zeeshan, Misha Khalid, Haseeb Javed Khan and Mahnoor Usman. Critical revision of the manuscript: Muhammad Hassan Waseem, Sania Aimen and Javed Iqbal.

## Ethics Statement

The authors have nothing to report.

## Consent

The authors have nothing to report.

## Conflicts of Interest

The authors declare no conflicts of interest.

## Supporting information


**Supplementary Figure S1:** Surgical site wound infection Forest plot.
**Supplementary Figure S2:** Walking speed Forest plot.
**Supplementary Figure S3:** Hospital for Special Surgery score Forest plot.
**Supplementary Figure S4:** Knee Society Score Forest plot.
**Supplementary Figure S5:** Lysholm score Forest plot.
**Supplementary Figure S6:** Oxford knee score Forest plot.
**Supplementary Figure S7:** WOMAC score Forest plot.
**Supplementary Figure S8:** Tegner score Forest plot.
**Supplementary Figure S9:** Postoperative pain leave‐one‐out sensitivity analysis.
**Supplementary Figure S10:** Hospital for Special Surgery score leave‐one‐out sensitivity analysis.
**Supplementary Figure S11:** Surgical site wound infection leave‐one‐out sensitivity analysis.
**Supplementary Figure S12:** Complications Funnel plot.
**Supplementary Figure S13:** Revision to Total Knee Arthroplasty Funnel plot.
**Supplementary Figure S14:** Range of Motion Funnel plot.
**Supplementary Figure S15:** Postoperative pain Funnel plot.
**Supplementary Figure S16:** Subgroup analysis for Complications.
**Supplementary Figure S17:** Walking speed subgroup analysis.
**Supplementary Figure S18:** KSS score.
**Supplementary Figure S19:** Oxford Knee Score.

## Data Availability

Data will be made available on reasonable request to the authors.
