# Supplementary material for: Comparison of Unicompartmental Knee Arthroplasty Versus High Tibial Osteotomy for Medial Knee Osteoarthritis: An Updated Meta‐Analysis of 56,000 Patients
Source: Orthop Surg. 2025 Jul 22;17(9):2499–513. doi: 10.1111/os.70049 (PMC12404872; doi:10.1111/os.70049)
Supplement: Supplementary file 1 — Supplementary Figure S1: Surgical site wound infection Forest plot. Supplementary Figure S2: Walking speed Forest plot. Supplementary Figure S3: Hospital for Special Surgery score Forest plot. Supplementary Figure S4: Knee Society Score Forest plot. Supplementary Figure S5: Lysholm score Forest plot. Supplementary Figure S6: Oxford knee score Forest plot. Supplementary Figure S7: WOMAC score Forest plot. Supplementary Figure S8: Tegner score Forest plot. Supplementary Figure S9: Postoperative pain leave‐one‐out sensitivity analysis. Supplementary Figure S10: Hospital for Special Surgery score leave‐one‐out sensitivity analysis. Supplementary Figure S11: Surgical site wound infection leave‐one‐out sensitivity analysis. Supplementary Figure S12: Complications Funnel plot. Supplementary Figure S13: Revision to Total Knee Arthroplasty Funnel plot. Supplementary Figure S14: Range of Motion Funnel plot. Supplementary Figure S15: Postoperative pain Funnel plot. Supplementary Figure S16: Subgroup analysis for Complications. Supplementary Figure S17: Walking speed subgroup analysis. Supplementary Figure S18: KSS score. Supplementary Figure S19: Oxford Knee Score. [file OS-17-2499-s001.docx]

| **Supplementary Table 1:** Detailed search strategies used in different electronic databases | | |
| --- | --- | --- |
| **Database** | **Search String** | **Results retrieved** |
| PubMed | ("Arthroplasty, Replacement, Knee"[Mesh] OR Arthroplasties, Replacement, Knee OR Arthroplasty, Knee Replacement OR Knee Replacement Arthroplasties OR Knee Replacement Arthroplasty OR Replacement Arthroplasties, Knee OR Replacement Arthroplasty, Knee OR Replacement, Total Knee OR Total Knee Replacement OR Knee Replacement, Total OR Knee Arthroplasty OR Arthroplasty, Knee OR Arthroplasties, Knee Replacement OR Knee Arthroplasty, Total OR Arthroplasty, Total Knee OR Total Knee Arthroplasty OR Arthroplasty, Replacement, Partial Knee OR Unicompartmental Knee Arthroplasty OR Arthroplasty, Unicompartmental Knee OR Knee Arthroplasty, Unicompartmental OR Unicompartmental Knee Replacement OR Knee Replacement, Unicompartmental OR Partial Knee Replacement OR Knee Replacement, Partial OR Unicondylar Knee Replacement OR Knee Replacement, Unicondylar OR Unicondylar Knee Arthroplasty OR Arthroplasty, Unicondylar Knee OR Knee Arthroplasty, Unicondylar OR Partial Knee Arthroplasty OR Arthroplasty, Partial Knee OR Knee Arthroplasty, Partial) AND ("Osteoarthritis"[Mesh] OR Osteoarthritides OR Arthritis, Degenerative OR Arthritides, Degenerative OR Degenerative Arthritides OR Degenerative Arthritis OR Osteoarthrosis OR Osteoarthroses OR Osteoarthrosis Deformans OR Arthrosis OR Arthroses) AND ("Osteotomy"[Mesh] OR Osteotomies) AND ("149[Mesh] OR Tibias) | 640 |
| Cochrane Library | (Unicompartmental Knee Arthroplasty) AND (Osteoarthritis) | 149 |
| ScienceDirect | (Unicompartmental Knee Arthroplasty) AND (Osteoarthritis OR Osteoarthritides ) AND (Osteotomy OR Osteotomies) AND (Tibia OR Tibias) | 745 |

| **Supplementary Table 2:** Newcastle-Ottawa scale for cohort studies quality assessment | | | | | | | | |
| --- | --- | --- | --- | --- | --- | --- | --- | --- |
|  | **Selection** | | | | **Comparability** | **Exposure** | | |
| **Studies** | **Representativeness of the exposed cohort** | **Selection of the nonexposed cohort** | **Ascertainment of exposure** | **Demonstration that outcome of interest was not present at the start of study** | **Comparability of cohorts based on the design or analysis** | **Assessment of outcome** | **Was follow-up long enough for outcomes to occur** | **Adequacy of follow-up of cohorts** |
| Broughton  1986 | * | * | * | * | * | * | * | * |
| Jefferson  1989 | * | * | * | * | * | * | * | * |
| Ivarsson  1991 | * | * | * | * | * | * |  | * |
| Weidenhielm 1992 | * | * | * | * | * | * | * | * |
| Weale  1994 | * | * | * | * | ** | * | * | * |
| Stukenborg 2001 | * | * | * | * | * | * | * | * |
| Borjesson  2005 | * | * | * | * | * | * | * | * |
| Takeuchi  2010 | * | * | * | * | * | * | * | * |
| Yim  2013 | * | * | * | * | ** | * | * | * |
| Tuncay  2015 | * | * | * | * | * | * | * | * |
| Petersen  2016 | * | * | * | * |  | * | * | * |
| Jeon  2017 | * | * | * | * | ** | * | * | * |
| Krych  2017 | * | * | * | * |  | * | * | * |
| Maxwell  2017 | * | * | * | * |  | * | * | * |
| Zhao  2017 | * | * | * | * | * | * | * | * |
| Cho  2018 | * | * | * | * |  | * | * | * |
| Ryu  2018 | * | * | * | * | * | * | * | * |
| Koh  2019 | * | * | * | * |  | * | * | * |
| Song  2019 | * | * | * | * | ** | * | * | * |
| Jacquet  2020 | * | * | * | * | ** | * | * | * |
| Chen  2020 | * | * | * | * | * | * | * | * |
| Hou  2020 | * | * | * | * |  | * | * | * |
| Zhang  2020 | * | * | * | * | * | * | * | * |
| Jin  2021 | * | * | * | * | * | * | * | * |
| Lin  2021 | * | * | * | * | * | * | * | * |
| Rodkey  2021 | * | * | * | * | * | * | * | * |
| Watanabe  2021 | * | * | * | * | * | * | * | * |
| Liu  2021 | * | * | * | * | * | * | * | * |
| Wyatt  2024 | * | * | * | * | * | * | * | * |
| Screpis  2023 | * | * | * | * | * | * | * | * |
| Teo  2024 | * | * | * | * | ** | * | * | * |
| Hoorntje  2023 | * | * | * | * | * | * | * | * |
| Zehir  2022 | * | * | * | * | * | * | * | * |
| Xu  2024 | * | * | * | * | ** | * | * | * |
| Lee  2021 | * | * | * | * | * | * | * | * |
| Neubauer  2023 | * | * | * | * | * | * | * | * |
| Ozgozen  2024 | * | * | * | * | ** | * | * | * |
| Karasavvidis 2024 | * | * | * | * | ** | * | * | * |
| Okimura  2023 | * | * | * | * | * | * | * | * |

| **Supplementary Table 3**: Egger’s Regression Test for Publication Bias | |
| --- | --- |
| **Outcomes** | **p-value** |
| Complications | 0.60792 |
| Revision to Total Knee Arthroplasty | 0.07306 |
| Range of Motion | 0.10399 |


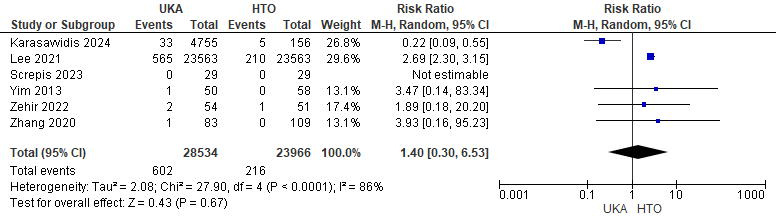


**Supplementary Figure 1:** Surgical site wound infection Forest plot


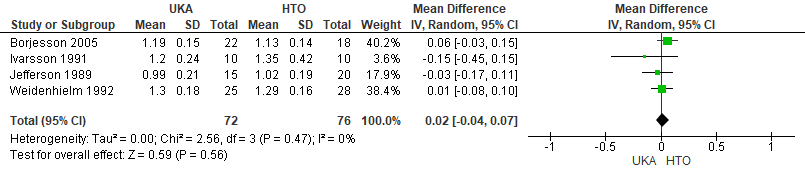


**Supplementary Figure 2**: Walking speed Forest plot


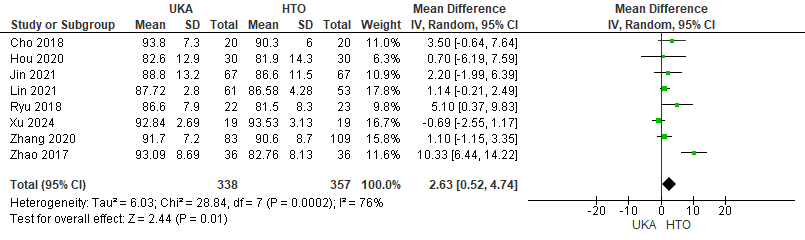


**Supplementary Figure 3:** Hospital for Special Surgery score Forest plot


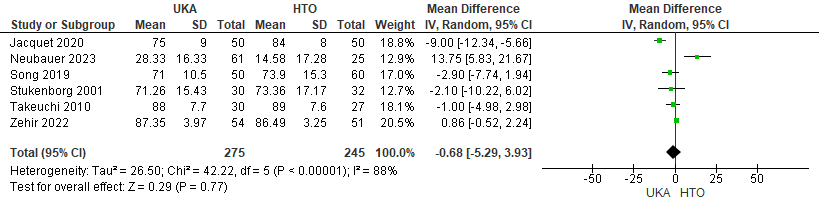


**Supplementary Figure 4:** Knee Society Score Forest plot

**
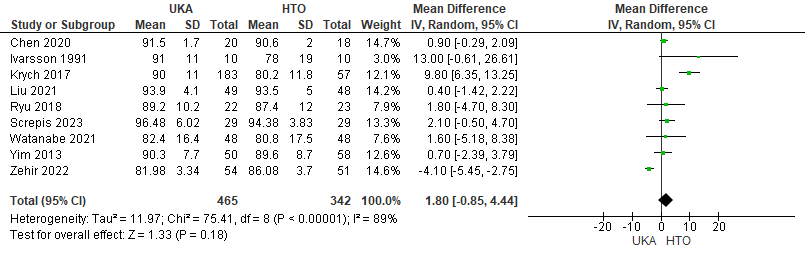
**

**Supplementary Figure 5:** Lysholm score Forest plot


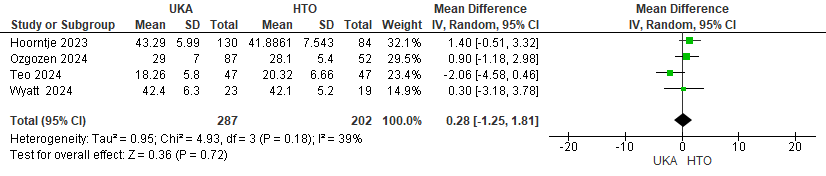


**Supplementary Figure 6:** Oxford knee score Forest plot


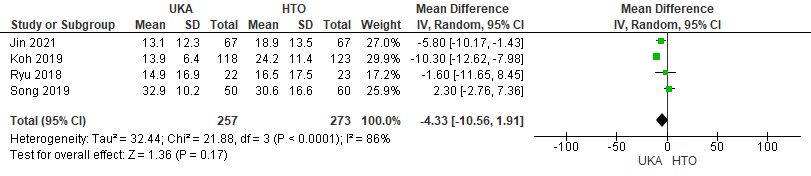


**Supplementary Figure 7:** WOMAC score Forest plot


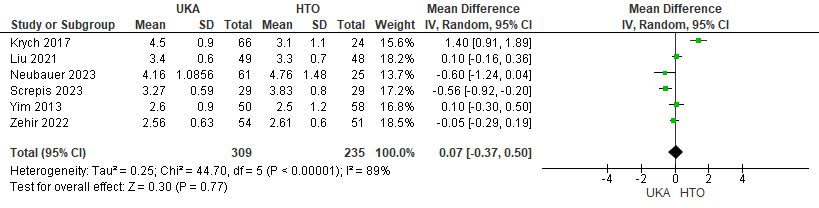


**Supplementary Figure 8:** Tegner score Forest plot


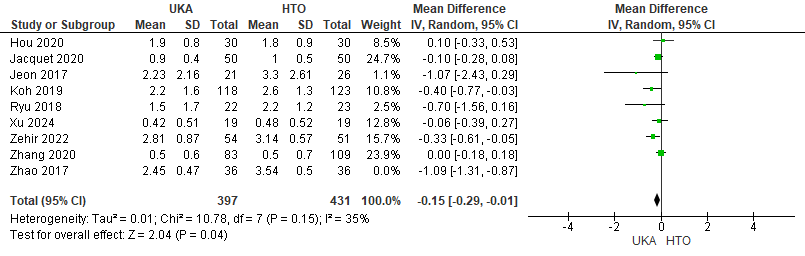
**Supplementary Figure 9:** Postoperative pain leave-one-out sensitivity analysis
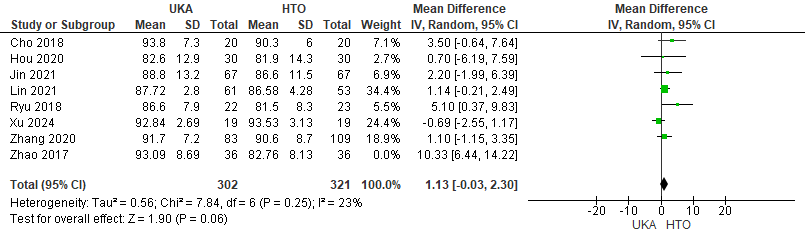
**Supplementary Figure 10:** Hospital for Special Surgery score leave-one-out sensitivity analysis


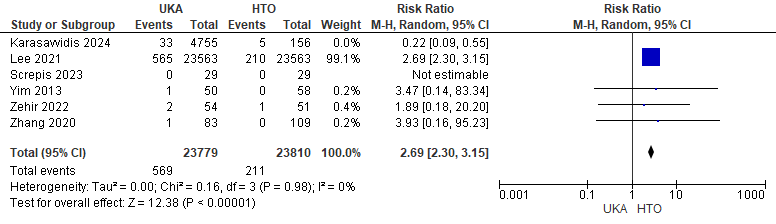


**Supplementary Figure 11:** Surgical site wound infection leave-one-out sensitivity analysis


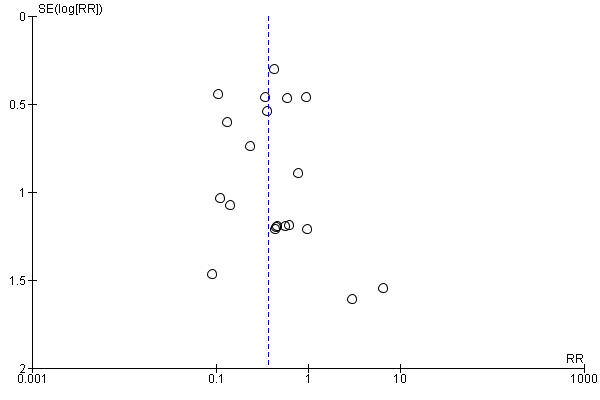


**Supplementary Figure 12:** Complications Funnel plot


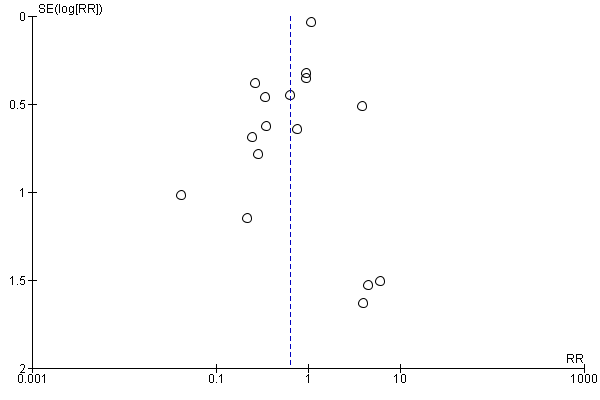


**Supplementary Figure 13:** Revision to Total Knee Arthroplasty Funnel plot


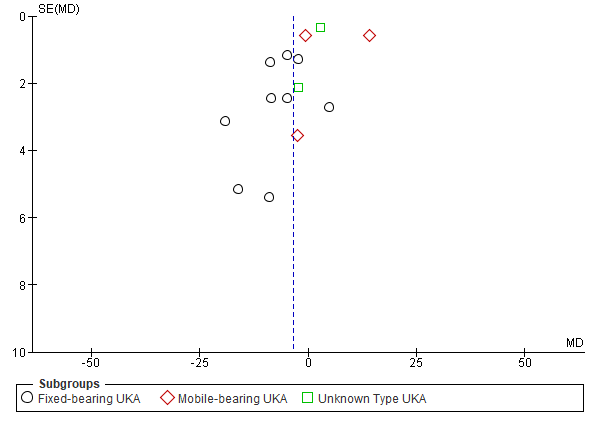


**Supplementary Figure 14:** Range of Motion Funnel plot


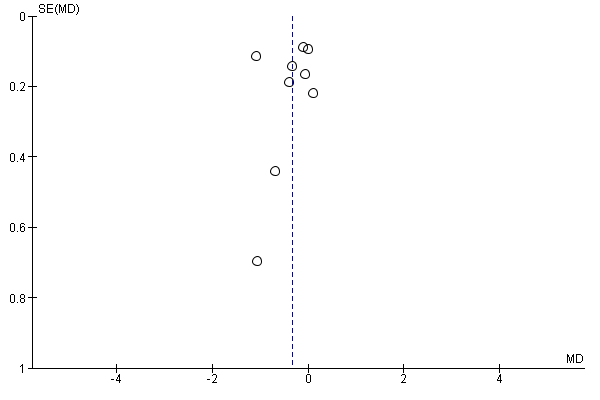


**Supplementary Figure 15:** Postoperative pain Funnel plot


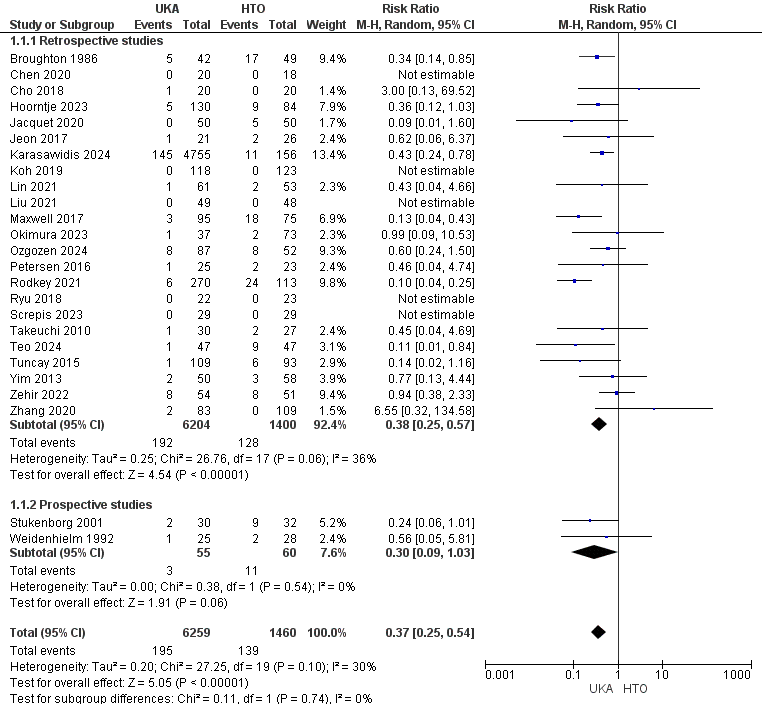


**Supplementary Figure 16:** Subgroup analysis for Complications

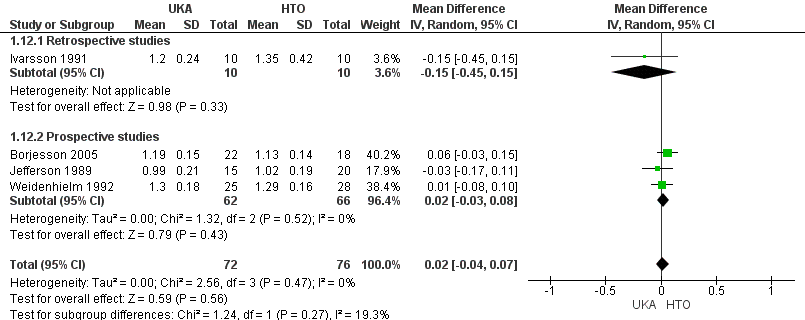


**Supplementary Figure 17**: Walking speed subgroup analysis


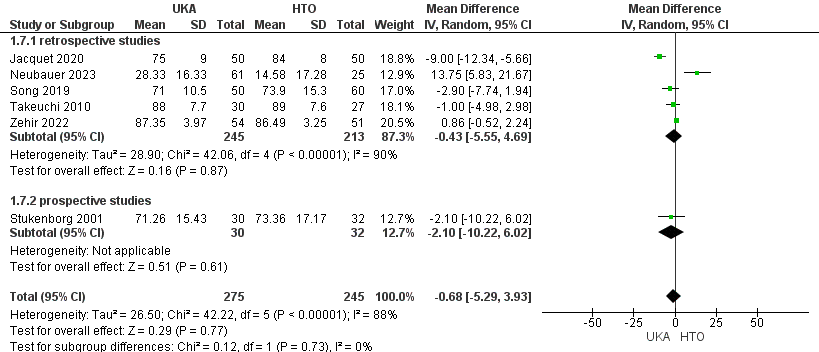


**Supplementary Figure 18: KSS score**

**
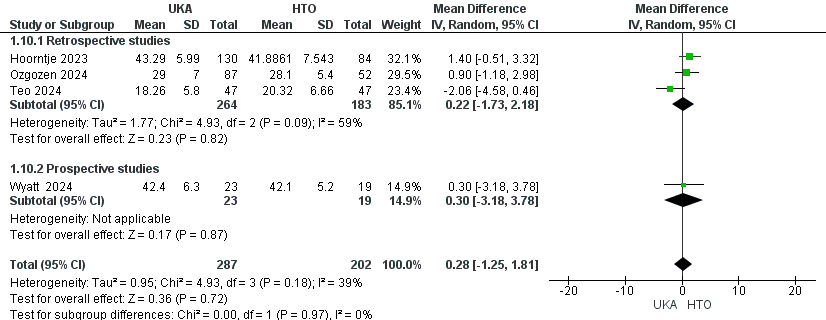
**

**Supplementary Figure 19: Oxford Knee score**
